# Case report: Presentations and cytokine profiles of inflammatory non-pulmonary COVID-19 and related diseases in children

**DOI:** 10.3389/fped.2023.1209772

**Published:** 2023-09-26

**Authors:** Yen-Chun Chao, Horng-Woei Yang, Lung Chang, Chih-Wen Tseng, Li-Ching Fang, Che-Sheng Ho, Hsin Chi, Kuender D. Yang

**Affiliations:** ^1^ Division of Cardiology, MacKay Children’s Hospital, Taipei, Taiwan; ^2^ Department of Pediatrics, MacKay Memorial Hospital, Taipei, Taiwan; ^3^ Department of Medicine, MacKay Medical College, Taipei, Taiwan; ^4^ Department of Medical Research, MacKay Memorial Hospital, Taipei, Taiwan; ^5^ Division of Infectious Disease, MacKay Children’s Hospital, Taipei, Taiwan; ^6^ Division of Allergy-Immunology-Rheumatology, MacKay Children’s Hospital, Taipei, Taiwan; ^7^ Division of Neurology, MacKay Children’s Hospital, Taipei, Taiwan; ^8^ Institute of Clinical Medicine, National Yang Ming Chiao Tung University, Taipei, Taiwan; ^9^ Department of Microbiology & Immunology, National Defense Medical Center, Taipei, Taiwan

**Keywords:** coronavirus disease 2019, non-pulmonary COVID-19, meningoencephalitis, acute necrotizing encephalopathy, multisystem inflammatory syndrome in children, SARS-CoV-2

## Abstract

The coronavirus disease 2019 (COVID-19) pandemic has evolved to dynamic waves of different SARS-CoV-2 variants. Initially, children diagnosed with COVID-19 presented pulmonary involvement characterized by mild diseases. In the later waves of the COVID-19 pandemic, life-threatening non-pulmonary inflammatory diseases such as (1) aseptic meningoencephalitis (ME), (2) acute necrotizing encephalopathies (ANE), and (3) multisystem inflammatory syndrome in children (MIS-C) have been reported, affecting the pediatric population. To alert timely identification and prevention of the life-threatening non-pulmonary COVID-19, we present the cases of ME, ANE, and MIS-C in terms of clinical manifestation, cytokine profile, and follow-up consequences. Based on the immunopathogenesis and risk factors associated with non-pulmonary COVID-19, we delineate strategies for an early diagnosis and treatment to reduce morbidity and mortality in children.

## 1. Introduction

Coronavirus disease 2019 (COVID-19) in children has dramatically changed from mild pulmonary diseases to the development of inflammatory encephalopathies and multisystem inflammatory syndrome in children (MIS-C) (1). The emerging subvariants of SARS-CoV-2 have demonstrated the ability to evade the immune response triggered by vaccinations and monoclonal antibodies (mAbs), leading to periodic re-emergence (2). The interactions among hosts, virus variants, and environments have caused different waves of the COVID-19 pandemic, with initial stages showing higher fatality and lower transmission and subsequent stages demonstrating higher transmission and lower mortality (3). Lower immunity with effective-limited vaccines and anti-virus medication has rendered children more susceptible to higher severity and mortality during the subsequent waves of the pandemic with non-pulmonary COVID-19 (4, 5).

Since October 2021, an increased rate of hospitalization among children with COVID-19 has been reported in the United States, Korea, Hong Kong, Singapore, and Taiwan (5–8). In the United States, the severity of MIS-C has significantly decreased over the pandemic waves from the Alpha, Beta, and Delta variants to the Omicron variants (9, 10). However, the prevalence of MIS-C associated with the Omicron variants has increased in Asia due to the lower exposure to COVID-19 among Asian children prior to the emergence of the Omicron pandemic (11). Children infected by the Omicron variants were prominently complicated with non-pulmonary diseases, including aseptic meningoencephalitis (ME), acute necrotizing encephalopathy (ANE), and MIS-C (12, 13).

Strict quarantine regulations on enforcing isolation protocols, use of face masks, and the practice of social distancing in Asia have resulted in a delay in the infection of children until the Omicron variants emerged (14). The Omicron variants had a higher transmission rate at home than that at school. In this study, we present the clinical features and immunopathogenesis of non-pulmonary COVID-19 in children to alert early diagnosis and treatment for prevention of morbidity and mortality.

## 2. Case presentations

Three cases of pediatric non-pulmonary COVID-19 were delineated based on their clinical presentation, and the underlying immunopathogenesis, early recognition, and prevention of potential fatal COVID-19 in children were discussed together. This study was approved by the Institutional Review Board of Mackay Memorial Hospital (21MMHIS363e), and the informed consents were obtained from the parents of the children.

### 2.1. Aseptic meningoencephalitis

**Figure 1:**
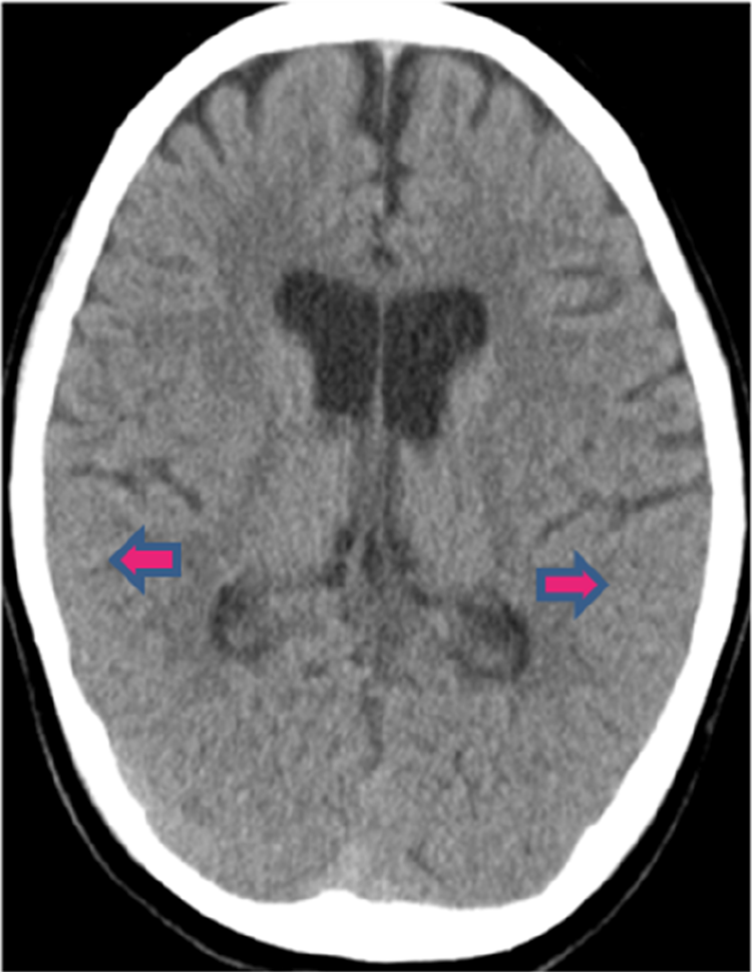
Brain CT scan of the ME patient. The CT scan revealed cerebral edema with sulcal effacement.

We present the case of a 6-year and 11-month-old girl with regular schedule of vaccination but had not received the COVID-19 vaccine. She contracted COVID-19 and experienced a recurring fever over a period of 6 days, accompanied by intermittent dizziness, headache, and conscious disturbance. She was admitted to the hospital due to poor feeding and vomiting. A physical examination of the patient revealed the presence of regular sinus tachycardia and clear breath sounds, with no observed skin rash or pathological reflex. A lumbar puncture procedure was performed, revealing a clear fluid and yielding a negative result on the Pandy test. However, WBC (lymphocytes) was measured at 8/mm^3^, RBC at 14/mm^3^, sugar at 67 mg/dl (blood sugar, 112 mg/dl), and total protein at 19 mg/dl. Measurements of cytokines in the blood and cerebrospinal fluid (CSF) were performed by multiplex beads array system from R&D Biosystems ((Minneapolis, MN, USA). The cytokine and chemokine profiles in CSF revealed higher levels of chemokines such as C-X-C motif chemokine 10 (CXCL10), also called 10 kDa interferon-gamma-induced protein (IP-10) 243.25 pg/ml and IL-8 32.32 pg/ml. The blood biochemistry analysis showed mild increases in alanine aminotransferase (ALT) levels at 33 IU/L (range 12–27 IU/L) and ammonia at 78 μg/dl (range 19–60 μg/dl). The blood IgG level was 797 mg/dl (range 490–1,610 mg/dl). The viral load in throat showed the cycle threshold (Ct) value of 25.6 on admission. The results of a brain computed tomography (CT) scan indicated the presence of cerebral edema with sulcal effacement (Figure 1). The hemoglobin level was 13.4 g/dl, platelet count was 271,000/mm^3^, WBC was 7,900/mm^3^ with 29% neutrophils, 58% lymphocytes (N/L ratio = 0.5), 10% monocytes, and 3% atypical lymphocytes. She was administered a daily dosage of 94 mg remdesivir and 20% mannitol (100 ml/12 h). The medications were discontinued after 3 days while the neurological symptoms and appetite improved, and the viral load decreased to the Ct value of 31.4. We excluded other common pathogens in CSF using the FilmArray assay, which detects 22 common respiratory tract pathogens (BioFire Diagnostics, SLC, UT, USA). The patient was discharged on the 6th day of admission. After 3 months of follow-up, she had fully recovered without any complication.

### 2.2. Acute necrotizing encephalopathies

This is the case of a 5-year and 2-month-old boy without pre-existing medical conditions. He has not received a COVID-19 vaccination. His aunt was diagnosed with SARS-CoV-2 infection 1 day prior to his onset of symptoms. He experienced upper respiratory symptoms for a duration of 1 day. He developed a high fever, loss of appetite, and decreased level of activity, and was then delivered to our emergency room (ER) for further evaluation and care. At the ER, the patient’s fever remained above 40°C, and a physical examination revealed enlarged tonsils and an overall appearance of illness. In addition, the patient had hyperactive bowel sounds and clear breath sounds. He experienced recurrent episodes of vomiting, developed seizures with loss of consciousness, and demonstrated an upward gaze of eyes and tonic posture. After receiving diazepam (0.3 mg/kg), he calmed down and was admitted to the pediatric ICU. A lumbar puncture found a clear fluid with a Pandy test result of 1+, 0 cell count, sugar of 75 mg/dl (blood sugar, 153 mg/dl), and a total protein of 147 mg/dl. The chemokines in CSF revealed dramatically higher levels of IP-10 (4,391.71 pg/ml) and IL-8 (1,863.2 pg/ml). The blood biochemistry data were examined and initially revealed normal levels of lactate dehydrogenase (LDH), creatine kinase (CK), and erythrocyte sedimentation rate (ESR), but a slight increase in the C-reactive protein (CRP, 1.31 mg/L) level and a higher lactate (34 mg/dl) level. Hemoglobin was 13.0 g/dl, platelet count was 188,000/mm^3^, and WBC was 8,800/mm^3^ with 63.9% neutrophils, 18.7% lymphocytes (N/L ratio = 3.4), and 11.7% monocytes. The levels of D-dimer, fibrinogen, LDH, aspartate aminotransferase (AST), ALT, and CK were found to be within normal limits, with 355 ng/ml, 217 mg/L, 255 IU/L, 56 IU/L, 25 IU/L, and 151 IU/L, respectively.

**Figure 2:**
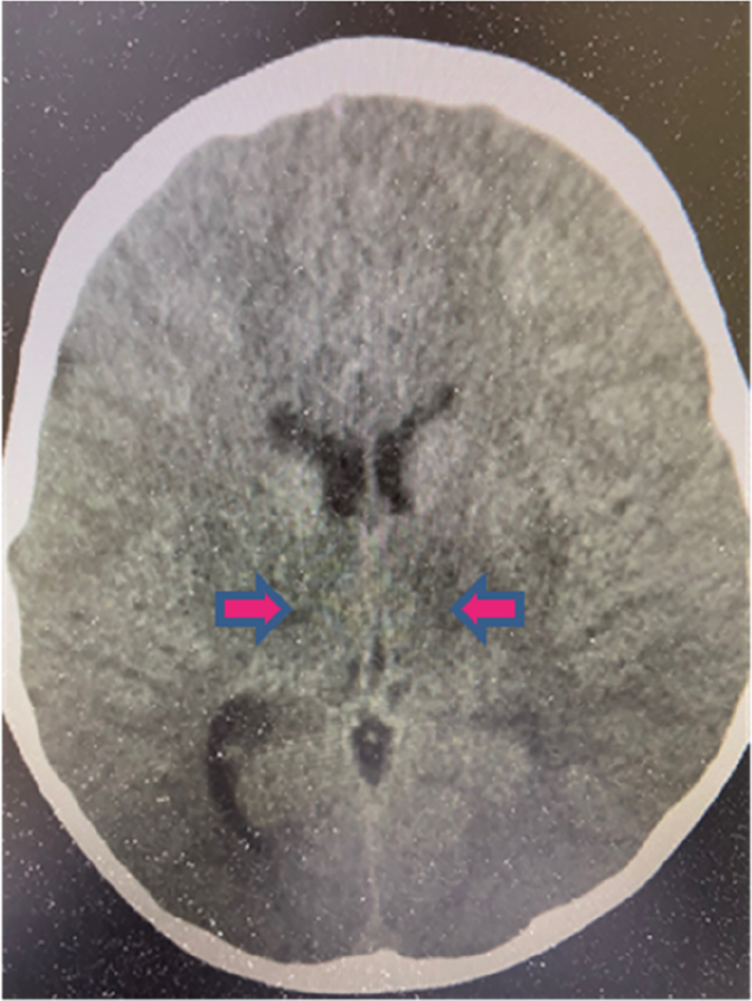
Brain CT scan of the ANE patient. The CT scan revealed hypodense edema involving bilateral thalami, dorsal pons, and midbrain.

The brain CT scan revealed the presence of hypodense edema affecting the bilateral thalami and dorsal pons extending to the midbrain (Figure 2). Despite administering 20% mannitol (100 ml/12 h) and dexamethasone (3 mg/12 h), his consciousness remained disturbed, and the levels of D-dimer, LDH, AST, ALT, and CK exhibited significant increases, reaching levels of 2,637 ng/ml, 3,055 IU/L, 1,830 IU/L, 3,041 IU/L, and 916 IU/L, respectively, 6 h following admission; fibrinogen concentration decreased to 151 mg/L, and platelets decreased to 120,000/mm^3^; total WBC was 10,200/mm^3^ with 82.1% neutrophils, 11.9% lymphocytes (N/L ratio = 6.9), and 6% monocytes; the CRP levels increased to 2.08 mg/dl. The patient was set to undergo hypothermia therapy at a temperature of 33.5 °C, in addition to receiving remdesivir (5 mg/kg/dose on day 1, followed by 2.5 mg/kg/day for a total of 5 days). Intravenous immunoglobulin (IVIG, 2 gm/kg), tocilizumab (12 mg/kg/dose), and methylprednisolone (30 mg/kg) pulse therapy were instituted. After 24 h, although the CRP levels reduced to 0.92 mg/dl and the lactate levels decreased to 18 mg/dl, the D-dimer, LDH, AST, ALT, and CK levels increased to 812 ng/ml, 1,168 IU/L, 1,487 IU/L, 1,270 IU/L, and 18,293 IU/L (creatine kinase-myoglobin binding, 265.2 ng/ml), respectively.

The patient was in a coma under hypothermia with subtle light reflex. The initial viral load present in the nasopharyngeal secretion was tested using an RT-PCR with a Ct value of 12.1. The remdesivir and hypothermia therapy were continued for 5 days. Following the successful recovery from hypothermia, the patient exhibited spontaneous respiration and involuntary movement, but she was in a semi-coma state. The abnormal biochemistry data gradually returned to normal ranges in 14 days, and the viral load of nasopharyngeal secretion gradually declined from a cycle threshold of 12.1, 16.8, 21.0, 30.4, and 32.6, weekly for 4 weeks. In the stable condition, the patient underwent a brain magnetic resonance imaging (MRI) study, which showed low signal intensity in the bilateral thalami, pons, and inferior part of the dorsal midbrain and multiple low signal intensities in the bilateral cerebral and cerebellar white matter. In addition, the splenium of the corpus callosum was also observed. The patient was transferred to a rehabilitation ward for physical therapy. The abnormal immune and biochemistry data significantly decreased in 1 week, but the patient developed cerebral palsy associated with leukomalacia of the periventricular, hypothalamus, and cerebellar regions in an MRI follow-up 3 months after the discharge.

### 2.3. Multisystem inflammatory syndrome in children

**Figure 3:**
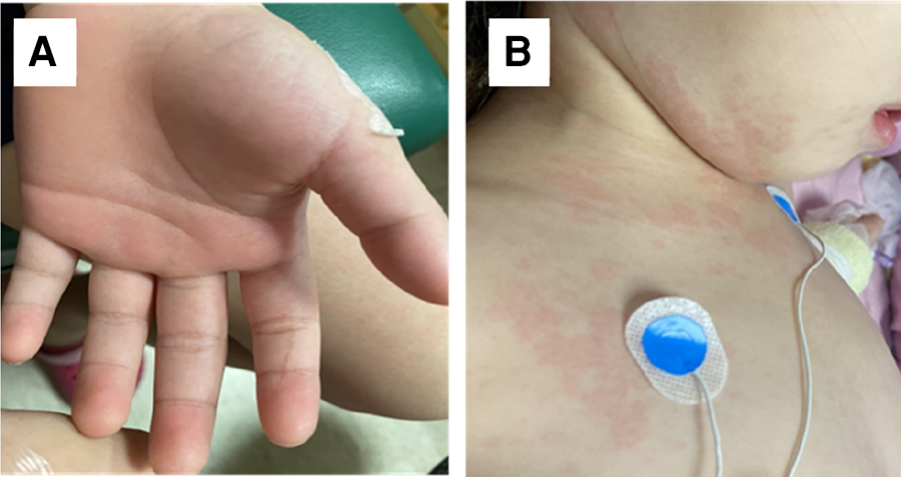
Skin rashes of the MIS-C patient. (**A**) Palm swelling on the patient with MIS-C, and (**B**) dysmorphic skin rashes on the MIS-C patient.

This is the case of a 4-year and 7-month-old girl who was healthy prior to exposure to COVID-19 and did not receive the COVID-19 vaccine. She was admitted due to a recurrent fever for 5 days. Historically, she contracted COVID-19 with mild fever and cough caused by a SARS-CoV-2 infection 4 weeks ago. Her parents were also diagnosed with COVID-19 6 days after her illness. She developed a high fever for 5 days and appeared with itchy and maculopapular rashes over her neck and face. She had visited an outpatient clinic due to her symptoms suggestive of a viral infection. The skin rashes gradually extended from the face and neck to the chest, abdomen, inguinal area, and limbs. Bilateral non-purulent conjunctivitis and swelling redness of palms and feet were also observed (Figures 3A,B). She exhibited fair level of activities with decreased appetite (50% of the usual amount) and urine output. Although there were no other symptoms reported such as headache, cough, dyspnea, chest pain, abdominal distress, vomiting, diarrhea, or convulsion throughout the course. Hypotension was noted on admission. The physical examination revealed skin rashes, swollen lips, non-purulent conjunctivitis associated with low blood pressure of 77/38 mmHg, and tachycardia at 148 beats per minute (BPM). The laboratory data results indicated anemia (Hb 9.9 g/dl), thrombocytopenia (platelets 132 K/μl), and lymphopenia [WBC 7,300/mm^3^ with 87% neutrophils, 8% lymphocytes (N/L ratio = 10.9)]. Serum albumin concentration was reduced to 2.4 g/dl. The inflammatory marker, C-reaction protein, was 29 mg/dl, procalcitonin was 15 ng/dl, and ESR was 61 mm/h. The coagulopathy indicators, D-dimer and fibrinogen, were 1,614 ng/ml and 583 mg/dl, respectively. The LDH and NT-proBNP levels were 310 IU/L and 12,200 pg/ml, but troponin-I and ferritin levels were within normal limits. The cytokine profiles in the plasma of the patient with MIS-C were measured by the multiplex bead array system from R&D Biosystems (Minneapolis, MN, USA), based on our previous study of Kawasaki disease model (15), and revealed the following results: IL-12 (371 pg/ml), IFNγ (946 pg/ml), IP-10 (129 pg/ml), and IFNα (17 pg/ml). The viral load in nasopharyngeal swab by an RT-PCR revealed a Ct value of 37.5. SARS-CoV-2 nucleoprotein antibody titers were 61 U/ml (range <10 U/ml) in serum. The IVIG (2 gm/kg) and methylprednisolone (30 mg/kg/day) pulse therapy were instituted after admission. Enoxaparin (1 mg/kg/day) and low dose of aspirin (5 mg/kg) were also prescribed due to high levels of D-dimer and fibrinogen. Initial heart echography revealed a decrease in the left ventricular (LV) ejection fraction at 46.6% with a dilated left atrium/LV. On the third day of admission, the patient developed hypotension with blood pressure of 69/36 mmHg and tachycardia of 125 BPM. Consequently, she was prescribed with an inotropic medication and dopamine (5 μg/kg/min). On the sixth day of admission, the LV ejection fraction increased to normal limits at 66%, and the blood pressure was normal, leading to the discontinuation of administering dopamine and low molecular weight heparin, enoxaparin (1 mg/kg/day). The patient was discharged on the seventh day of admission while being prescribed a low dose of aspirin (5 mg/kg). In a follow-up after 3 months, the patient recovered smoothly with the presence of mild anemia. No evidence of coronary arterial lesion was detected on cardio-sonography.

## 3. Discussion

### 3.1. Pathogenesis of non-pulmonary COVID-19 diseases

#### 3.1.1. Pathogenesis of aseptic meningoencephalitis

The pathogenesis of aseptic COVID-19 ME could be related to four different mechanisms: (1) virus invasion to the brain via the olfactory nerve, (2) hematogenous dissemination of virus, (3) immune-mediated cytokine storm, and/or (4) COVID-19-associated leukocyte activation leading to inflammation (12, 16). It is important to differentiate the pathogenesis of the virus invasion from the immune-mediated mechanisms to be able to choose an appropriate treatment involving either an anti-virus or anti-inflammation treatment. This patient with aseptic ME presented here may potentially be associated with immune-mediated inflammation, as indicated by the absence of common pathogenic microbial nucleic acids in CSF detected by FilmArray assay and relatively low viral load on the throat secretion. To provide evidence for the immune-mediated mechanism, we have demonstrated the higher levels of IP-10 and IL-8 in CSF.

#### 3.1.2. Pathogenesis of ANE

ANE associated with COVID-19 is not a novel disease entity; in fact, ANE usually occurs to children after an infection with influenza, rotavirus, enterovirus, or herpes virus (17, 18). In Asia, ANE is prominently related to an influenza infection associated with a sudden onset of high fever and intractable seizure, and progresses to necrosis of deep brain neurons with a high fatality within a few days (17, 18). Although the prevalence of ANE is much lower in Caucasians, it could occur to subjects with mutations in the RAN binding protein 2 (*RANBP2*) gene, named as ANE type 1 (*ANE1*), which can be familial and recurrent (18). Many other genes involved in the metabolism or immunity have been linked to ANE, including neuronal sodium voltage-gated channel alpha subunit 1 (*SCN1A*), carnitine palmitoyltransferase II (*CPT2*), and human thiamine transporter 2 (h*THTR2*) (18). Both *CPT2* and h*THTR2* mutations are very sensitive to febrile infections in which mitochondrial dysfunction is linked to hyperinflammation (17, 18). We treated this patient with a combination of IVIG, pulse methylprednisolone, and hypothermia (33.5 °C) therapies in 24 h. This treatment resulted in a decrease in the lactate and CRP levels, but it did not rescue the severe neurological sequela. The patient with ANE reported in this study was hospitalized during the outbreak of Omicron B1.1.529. The variant has been reported to cause non-pulmonary COVID-19 with a higher hospitalization rate and fatality in children (14). The Omicron-derived variants in Taiwan have primarily evolved into BA.2.75, BQ.1, and XBB, and few variants with BQ.1.1, BF.7, BA.2.75.2, CH.1.1, and XBF.

#### 3.1.3. Pathogenesis of MIS-C

The clinical manifestations of MIS-C are somehow similar to KD, showing augmented inflammatory symptoms as elevated CRP, persistent high fever, non-purulent conjunctivitis, skin rashes, and cardiovascular events. In contrast, MIS-C patients had an older age onset, a history of recent COVID-19, coagulopathy with higher D-dimer, and thrombocytopenia (15). KD is prevalent in Asia, but MIS-C is initially reported in Western countries subsequent to the COVID-19 pandemic, although the prevalence and severity of MIS-C had significantly declined from Alpha to Delta variants in Western countries (9, 10). However, there has been a surge in the prevalence of Omicron variants across Asia (10). We have here further compared the different cytokine and chemokine profiles in the blood between MIS-C and KD, showing significant increases in IL-12 and IFNγ levels in MIS-C in comparison with those in KD. However, the IL-1ß, IL-6, IL-8, TNFα, IP-10, and IL-10 levels were not considerably different between MIS-C and KD (data not shown). This finding suggests that cytokine storm between KD and MIS-C contributes to the same phenotypes, but somehow different immune alterations. MIS-C exhibits similarities to the KD shock syndrome (KDSS), which is frequently found in female Hispanos with older age and more frequently found with shock syndrome and higher fatality (15). The current mortality of MIS-C in Omicron variants is approximately 1.0% with early detection and treatment with IVIG and pulse methylprednisolone, and add-on anti-inflammatory treatment of anakinra, anti-TNF, or anti-IL-6 when refractory, or add-on anticoagulants when thromboembolism is present (9, 10). This suggests that early detection of MIS-C is crucial for a timely intervention, rather than delayed treatment due to atypical presentations.

### 3.2. Host factors on the development of non-pulmonary COVID-19 in children

Children were spared from severe pulmonary COVID-19 in the beginning of the pandemic. No deaths were reported in East Asia prior to October 2021 (5–8). During the Omicron pandemic, a certain portion of children died of non-pulmonary COVID-19 (4–6, 18). In addition to virus variants, host factors such as age, race, and genetic variants contribute to the immunopathogenesis for the prevention and immunotherapies of different non-pulmonary COVID-19 in children (Table 1). Moreover, children with cardiovascular or neurological comorbidities are associated with a higher risk to post-COVID-19 complications (19).

**Table 1:** Host factors, immunopathogenesis, and prevention of non-pulmonary COVID-19 in children.

Non-pulmonary COVID-19	Aseptic meningoencephalitis	Acute necrotizing encephalitis	Multisystem inflammatory syndrome in children
Immunopathogenesis	1. Age-associated immature immunity	1. Race: Asians >Caucasians	1. Race: Blacks, Asians
	2. Low neutralizing Abs with higher viral load	2. Genes: HLA, *RANBP2*, *CPT2*, *SLC19A3*, *SCN1A*	2. Genes: *TRBV11-2*, etc., linked to Th1 cytokine storm: *IL-12 p70*, *IFNγ*, etc.
	3. Interruption of BBB	3. Metabolism linked to hyperinflammation	3. Autoimmune vasculitis
Prevention and immunotherapies	1. Prophylactic vaccines	1. Screen genetic variants of hyperinflammation	1. Early IVIG and pulse therapy
	2. Passive immunization via maternal vaccination	2. Mitochondrial cocktails	2. Anti-cytokine storm therapy or anti-thrombosis treatment

Abs, antibodies; BBB, blood–brain barrier; *CPT2*, carnitine palmitoyltransferase II; *RANBP2*, RAN binding protein 2; *SLC19A3*, solute carrier family 19 member 3; *SCN1A*, sodium voltage-gated channel alpha subunit 1; *TRBV11-2*, T cell receptor beta variable 11-2.

It is unclear how genetic variant(s) is (are) involved in the pathogenesis of non-pulmonary COVID-19. There are certain genetic variants associated with ANE including *RANBP2*, *HLA*, *CPT2*, *SCN1A*, and *SLC19A3* in the COVID-19-associated ANE. *CPT2* is sensitive to fever associated with lower enzyme activity, contributing to mitochondrial dysfunction characterized by oxidative stress and inflammation. Mutation of *SCN1A* is associated with febrile seizure, and *SLC19A3* is associated with defective thiamine transportation for mitochondrial energy supply via the acetyl-CoA metabolism (Table 1) (18).

### 3.3. Special considerations on the prevention of non-pulmonary COVID-19 in children

#### 3.3.1. Prophylactic vaccines and passive immunization

In consideration of the safety of COVID-19 vaccines for children, especially infants, the emergency use authorization (EUA) of child COVID-19 vaccines was issued in the later waves of Delta and Omicron variants. A passive immunization utilizing specific mAbs was also authorized for children and infants in the later stages of the COVID-19 pandemic. However, the SARS-CoV-2 has evolved into immune escape of vaccines and mAb. Fortunately, the vaccines developed for the Wuhan strain, although not providing complete protection against SARS-CoV-2 infection, are useful for reducing morbidity and mortality of COVID-19. It is important to ensure the effective neutralizing antibodies for newborns and preterm babies via transplacental transportation of IgG from pregnant women with full vaccination before the third trimester (Table 1). Furthermore, in event of immune evasion of COVID-19 vaccines and mAb in children, the concept of herd immunity in the household remains very important. This is due to the fact that family contacts mediate higher transmission of Omicron variants compared with school contacts. Evidence also suggests that the COVID-19 vaccination should protect against developing MIS-C, one should also notice the potential occurrence of MIS-C after COVID-19 vaccination, which has been referred to as MIS-V (9, 10, 19, 20). Whether the COVID-19 vaccination also protects against ANE remains unknown.

#### 3.3.2. Targeting cytokine storm and altered immune reaction

Immunotherapies aimed at mitigating cytokine storm or inflammation by anti-IL-6 receptor, tocilizumab (ACTEMRA), or Janus kinase inhibitor (JAKi) such as Olumiant (baricitinib) have been approved for treating COVID-19 in people suffering from adult respiratory distress syndrome (ARDS) to reduce morbidity and mortality. Tocilizumab can be administered to children under age 12, but not Olumiant yet. Further studies are needed to investigate whether these anti-cytokine storm regimens could rescue children with non-pulmonary COVID-19 encephalopathies.

#### 3.3.3. Immunomodulation of non-pulmonary COVID-19 hyperinflammation

The immunopathogenesis of non-pulmonary COVID-19 in children is different from that of ARDS in adults and requires different immunotherapies. For individuals diagnosed with ANE, early methylprednisolone pulse therapy and hypothermia therapy should be initiated within 24 h (18). Children with ANE due to fever sensitivity or mitochondrial dysfunction should be administered with mitochondrial cocktails containing biotin, thiamine, L-carnitine, and CoQ10 within 24 h. Children with MIS-C require early administration of IVIG and methylprednisolone pulse therapy.

## 4. Limitations of the report

There are some limitations of this series of case report: (1) the host genome sequence analysis of potential susceptible genes in non-pulmonary COVID-19; (2) the exact SARS-CoV-2 variants in each case with ME, ANE, and MIS-C were not analyzed; (3) the literature review for the discussion is limited by the journal’s policy of allowing a maximum of 20 references.

## Data Availability

The original contributions presented in the study are included in the article, further inquiries can be directed to the corresponding authors.
